# Palmer’s Patent Leg in London—Letter from Mr. Palmer

**Published:** 1851-12

**Authors:** 


### *Palmer's Patent Leg in London—Letter from Mr. Palmer.*

Office of Palmer's Patent Leg,
30 Regent St., London, Oct. 1st, 1851.

To the Editor of the Boston Medical and Surgical Journal.

Dear Sir,—Not wishing to write for public criticism unless I have sufficient matter of *fact* to arrest the attention of the reader, I have delayed sending my second letter to your esteemed Journal until the present time; and as I am cheerfully expecting to step from the steamer into Boston very soon, this will be the last which I shall have the honor of addressing to you till I have said "good-bye" to our esteemed "cousin" John Bull, Jr., Esq.

I have informed you of my reception in London—of my unexpected success up to the time of writing in July, and that I was about to open a manufactory here. My progress since has been more remarkable, and I now find myself in the midst of a business the extent of which yon could scarcely believe possible. I am actually *run down* by the lame—and I might add, "the lazy." At the time you left London, the licensees for the manufacture here were Bigg & Son, of Leicester Square; but foreseeing a demand for the invention much beyond their calculation or readiness properly to supply, and having much better opportunities to dispose of the patent, I purchased their interest in the same, and have made a more satisfactory arrangement with other parties. The manufactory is now in successful operation, with genuine Yankees at its head, and the sale has commenced under the most auspicious circumstances. Among the many distinguished patrons we have already secured, is the very eminent Guthrie, who has to-day sent me the following certificate:

"4 Berkley St., Berkley Square, Sept. 30, 1851.

"Sir,—I have no hesitation in stating, according to your desire, that I consider your Patent Artificial Leg to be the best invention I have yet seen—the most useful, and the least distinguishable from the natural limb.

I am, Sir, your very obedient servant,

"Mr. Frank Palmer. G. J. Guthrie."

Applications are now received for about $8000 worth of limbs, and among the applicants are many very distinguished persons — one being General Sir William Harris, of this city; another, the famous General Doll, of the Austrian army; and another, General Talbot, of Aix la Chapelle, Belgium.

The first limb manufactured here was for a distinguished lady — the wife of Dr. Turner, of Kensington. Dr. T. is an eminent and rising surgeon, and a truly clever fellow. I have been, by Dr. and Mrs. Turner, most cordially welcomed at their house; where, in social parlance, undisturbed by the tyrant *fashion*, or the moustached sycophant *etiquette*, a genuineness of soul has been manifested; and it is with a feeling of pride that I mention them as among the choicest of my friends in London.

But I must not go on to enumerate the esteemed names of those who have received me with friendly salutations; and if, on the other hand, I have met a *few* who deemed it becoming to appear with the auricular organ essentially elongated, I will not waste my ink in writing of them. I am aware that my tale is quite unlike that of many of my countrymen, and that I may be charged with superior gullibility, which allows our cousin John to *cozen* me into an absurd belief, as he lays on the mortar with an *exceedingly large trowel!* Be it so; even then would it be yankee *cuteness* to remonstrate against excessive coating, if each coat applied has large pockets well lined with the choicest fabric from the — Bank of England? But I am not inclined to look at things in this light; and that fulsome flattery is found in different circles from those in which it has been my good fortune to move. I am constrained to state that my personal experience has been such as to demand that I speak well of the English people. And I am convinced that any of my countrymen, who have inventions of *merit*, need not fear to present them in London. There may be seeming inattention at first, or severe criticism, but the things of true merit will, eventually, be well received. An American will, it is true, meet with much to amuse and surprise him, while offering his "notions" to an English public; and if a zany of one class attacks him in a manner calculated to excite his "dander," before he can become "furious" a zany of another class will have him convulsed in a fit of laughter. Had I the pen of a certain English author who was received by a generous American people with more attention than he deserved, I might, in retaliation for his shabby treatment of us, tell you many a tale of English impressibility, which would excite the risibility even of a chaplain of the Inconsolable Society. But I come not to England to make sport of her unfortunate poor, or of the more unfortunate wealthy whose treasures are found only in their coffers; neither did I come to traduce the character of her intelligent inhabitants, who receive me with hospitality, even though they show me more respect than is due so humble an individual. But I will narrate a single incident of the class at which I have hinted, and that shall be for the especial benefit of the man who burst his straps with laughter, because a dozen urchins of the Boston Ragged School pursued him for his *autograph*. And, in passing, I will suggest that what caused the boys to press about him for a specimen of his hand-writing, was, they took him for a *village writing master, from away down East*, and thought he might (as he did) cut some flourish for their amusement — such, for instance, as a goose.

On Sunday last the leg excitement was intense, as you may suppose, after the potential article in the London times had been digested by the learned persons who throng the transept and naves of the Crystal Palace on the *half*

*crown days.* In addition to what the Times, the Art Journal, the Illustrated News, the Chronicle, and many other of the leading London papers, had said about the leg, Punch, last week, under the caption of "Palmer's Legs," gave a cut of himself preparing for amputation, to enable him to avail himself of the improvements in person, and inserted a long article below it as his leader, in which, among many other things, he said, "Indeed, Mr. Palmer thinks he can perfect his invention, and construct whole bodies which will perform perfectly, which will execute *pirouettes*, *entrechats*, and so forth; sigh, grin, pout, leer and ogle as well as the very best *coryphees.* The *corps de ballet* is much excited, and Mr. Lumley is in treaty for six dozen of these *danseuses*." Such an announcement aroused the fashionables *en masse*, and the crowd that pressed around me on Saturday, evidenced that, truly as the *Times* (a word in which is magic) had been believed, Punch, also, had been accredited. A lady present, who had been a constant visitor in the Crystal Palace since the opening in May, while inspecting the leg, said, with earnestness, "What a pity it is this great invention was not here sooner — I saw in the papers that it had only been here about a week." I replied, "These limbs have been here in this place, as you now see them, since two weeks prior to the opening of the exhibition." "Why," continued she, "have I not seen them before, as I have passed here so often?" "The reason is this, madam — the first four months of the exhibition, the *Times* and *Punch* told you that there was nothing to be seen in the 'American Prairie,' and so you saw nothing! Now, the Times tells you that your 'cousins are as pre-eminent in leg-making as in yacht-building,' and Punch declares that Mr. Lumley is in treaty for a quantity of patent legs to supply the *corps de ballet*, and you see them." Hesitating a moment, the lady replied, "I suppose it is so." And yet she was not one of those *poor mortals* who are compelled to

"Stitch, stitch, stitch, from weary chime to chime,"

But was

"Dressed as the nobles dress,
In robes of silver and gold;
Silks and satins and costly furs,
In many an ample fold."

Now, I ask, who appeared best, the ragged fellows who chased "Boz," thus evincing a desire to *learn something* of the man they mistook for an English gentleman; or this lady, who looked for four months at a case of full-sized legs, and did not see them till Punch said they were better than natural ones in opera or polka? If, this week, Punch should announce that Mr. Lumley's order has been executed, that lady would be seen nightly at her Majesty's theatre, squinting at the beautiful patent *danseuses* through a pair of Colt's revolvers.

But enough of this, and I leave the subject by informing you that "the American Leg," in competition with some thirty of the best European manufacture, among which are the best Paris limbs, has received the award of the only "*prize medal*" in this department of mechanism in the Crystal Palace. I have received renewed invitations from the faculty of Paris, and go there (for a few days only) on Saturday next. M. Charriere, and M. Luer, the renowned surgical instrument and limb makers of Paris, have both applied for my French patent.

The great exhibition will close on the 11th inst. I saw the grand *opening*

on the first of May, and shall see the grand *closing*. You have recently learned that the "American Prairie" of the Crystal Palace has now become the center of attraction, and that after all the sport made of our *notions*, Jonathan will embark for home, with his *yacht well loaded with medals!* In fact, I shall not be surprised if he gets rather more than his share of the honors. At any rate, the London Times has frankly and manfully declared that the yankees "have taken the shine out of them," and has made the most ample reparation for all it said disparagingly of our representation in the Crystal Palace during the early part of the exhibition. But for one, I am little inclined to find fault with the course the Times has pursued, and a little *rubbing up* did us no harm. In fact, there were many *rusty notions* in our division, which *required* rubbing, and then our best articles could not possibly have been appreciated till explained, and many of them tested in competition with others. Yachts had to be run — grain cut — furrows turned — locks picked which had defied the skill of man for a quarter of a century— pistols fired, and legs probed until they could be decided to be *artificial*, before the worth of our inventions about which most has been said, but assure you there are many other splendid triumphs of American ingenuity, as the list of awards will soon tell. But all is now good-natured rivalry. The Times has proposed to "shake hands" with us, and separate as good and affectionate cousins. This is fair — is manly— is *fraternal*. And bidding the leviathan of papers and its esteemed and clever editors, together with numerous Engltsh friends, a kind good-bye, I shall step on board the steamer Europa, on the 18th inst., and shall "hooray" for the Cunard line until I am landed safe in Boston, whence I shall proceed immediately home to Philadelphia to spend the winter.

I am, sir, your most obedient servant, B. F. PALMER.

